# Commentary: Editorial bullying: an exploration of acts impacting publication ethics and related environment

**DOI:** 10.3389/frma.2026.1772427

**Published:** 2026-02-02

**Authors:** Afsaneh Shirani

**Affiliations:** 1 Saint Luke's Marion Bloch Neuroscience Institute, Kansas City, MO, United States; 2 Department of Neurology, University of Missouri-Kansas City, Kansas City, MO, United States

**Keywords:** coercive citation, conflict of interest, editorial bullying, editorial misconduct, publication ethics

## Introduction

I read with great interest the mini-review by Javed et al. (2024) examining editorial bullying and misconduct within scientific publishing. By shifting attention from author-centered misconduct to abuses of editorial authority, the authors highlight a critical but often under-discussed threat to research integrity: the subtle ways power dynamics within journals can shape scholarly behavior, distort the scientific record, and undermine trust in the publication process.

One form of editorial misconduct that aligns closely with the concerns raised in this review—but deserves more explicit recognition—is coercive citation (Committee on Publication Ethics (COPE), 2024; Wren et al., 2019), particularly when authors are required to cite work authored by a journal's editor-in-chief or editorial leadership as a condition of submission or review. While the authors discuss intimidation, biased decision-making, and misuse of editorial power, coercive citation represents a quieter yet equally consequential mechanism through which influence may be exerted.

## Coercive citation as a form of editorial misconduct

An illustrative example of coercive citation involves situations in which authors are asked to cite a guideline or publication authored by a journal's editorial leadership as a condition of submission or review. In one such example, a commentary was submitted to a peer-reviewed journal in the field of medicine and surgery. As part of the submission process, the author was required to complete a checklist aligned with a recently published guideline for the transparent reporting of artificial intelligence in scientific publications. While affirming compliance with transparency and reporting standards was appropriate, the author was informed that citation of the guideline's associated publication—first authored by the journal's editor-in-chief—was also mandatory within the body of the manuscript, despite the submission being a letter to the editor. The author indicated willingness to complete and submit the checklist; however, obligating citation of a guideline whose first author is the journal's editor-in-chief raised ethical concerns. The journal emphasized that the citation was mandatory and that the manuscript would not be reviewed without it, ultimately leading to withdrawal of the submission. Review of recently published articles in the same journal suggested that the guideline had been cited in contexts where it appeared totally irrelevant, seemingly to satisfy this enforced requirement.

This experience reflects several elements described by Javed et al., including misuse of editorial authority, intimidation through conditional review, and practices that compromise ethical norms. Requiring authors to cite specific articles—particularly those authored by editorial leadership—creates an inherent conflict of interest and risks transforming citation from a scholarly act into an instrument of compliance. Such practices may artificially inflate citation metrics, distort impact factors, and erode the principle that citations should be driven by relevance and scientific merit rather than editorial mandate.

Importantly, widely accepted reporting frameworks such as CONSORT, PRISMA, and STROBE have achieved legitimacy through community consensus, transparent development, and voluntary uptake. Emerging guidelines, however valuable, should follow a similar trajectory of ethical dissemination. Mandating citation as a prerequisite for submission—especially when relevance is marginal—crosses the boundary from guidance to coercion. As Javed et al. note, editorial behaviors that suppress dissent or exploit power dynamics threaten the integrity of scholarly exchange; coercive citation fits squarely within this conceptual framework.

## Discussion

Within the taxonomy proposed by Javed et al., coercive citation can be conceptualized as a form of editorial bullying rooted in the misuse of positional authority and exploitation of power asymmetries in the publication process. When citation is imposed as a condition for review or consideration—particularly when involving work authored by editorial leadership—it aligns with the authors' descriptions of biased decision-making, intimidation through conditional gatekeeping, and conflicts of interest that compromise editorial integrity. In this sense, coercive citation represents a subtle but consequential mechanism through which editorial influence may shape scholarly behavior, extending the framework outlined by Javed et al. to include citation-based coercion. While the prevalence of coercive citation is difficult to quantify due to the confidential and anonymized nature of editorial processes, its ethical significance lies in the misuse of editorial authority rather than in its frequency.

Addressing editorial bullying requires not only awareness but structural safeguards. In addition to the authors' call for education and professionalism, journals might consider explicit policies prohibiting mandatory self-citation by editors, independent oversight of editorial practices, and anonymous mechanisms for authors to report coercive behaviors without fear of retaliation. Transparency in editorial decision-making must extend beyond authorship disclosures to include how citation expectations are communicated and enforced.

I commend Javed et al. for bringing critical attention to editorial misconduct. Expanding this conversation to include coercive citation practices may further strengthen efforts to protect academic integrity and ensure that scientific publishing remains a forum for open, evidence-based discourse rather than hierarchical compliance.
